# Immunization with a Recombinant Vaccinia Virus That Encodes Nonstructural Proteins of the Hepatitis C Virus Suppresses Viral Protein Levels in Mouse Liver

**DOI:** 10.1371/journal.pone.0051656

**Published:** 2012-12-17

**Authors:** Satoshi Sekiguchi, Kiminori Kimura, Tomoko Chiyo, Takahiro Ohtsuki, Yoshimi Tobita, Yuko Tokunaga, Fumihiko Yasui, Kyoko Tsukiyama-Kohara, Takaji Wakita, Toshiyuki Tanaka, Masayuki Miyasaka, Kyosuke Mizuno, Yukiko Hayashi, Tsunekazu Hishima, Kouji Matsushima, Michinori Kohara

**Affiliations:** 1 Department of Microbiology and Cell Biology, Tokyo Metropolitan Institute of Medical Science, Setagaya-ku, Tokyo, Japan; 2 Division of Hepatology, Tokyo Metropolitan Komagome Hospital, Bunkyo-ku, Tokyo, Japan; 3 Transboundary Animal Diseases Center, Joint Faculty of Veterinary Medicine, Kagoshima University, Korimoto, Kagoshima, Japan; 4 Department of Virology II, National Institute of Infectious Diseases, Shinjuku-ku, Tokyo, Japan; 5 Laboratory of Immunobiology, Department of Pharmacy, School of Pharmacy, Hyogo University of Health Sciences, Chuo-ku, Kobe, Japan; 6 Laboratory of Immunodynamics, Department of Microbiology and Immunology, Osaka University Graduate School of Medicine, Suita, Osaka, Japan; 7 Chemo-Sero-Therapeutic Research Institute, Okubo, Kumamoto, Japan; 8 Department of Pathology, Tokyo Metropolitan Komagome Hospital, Bunkyo-ku, Tokyo, Japan; 9 Department of Molecular Preventive Medicine, School of Medicine, University of Tokyo, Bunkyo-ku, Tokyo, Japan; University of Montreal, Canada

## Abstract

Chronic hepatitis C, which is caused by infection with the hepatitis C virus (HCV), is a global health problem. Using a mouse model of hepatitis C, we examined the therapeutic effects of a recombinant vaccinia virus (rVV) that encodes an HCV protein. We generated immunocompetent mice that each expressed multiple HCV proteins via a Cre/*loxP* switching system and established several distinct attenuated rVV strains. The HCV core protein was expressed consistently in the liver after polyinosinic acid–polycytidylic acid injection, and these mice showed chronic hepatitis C-related pathological findings (hepatocyte abnormalities, accumulation of glycogen, steatosis), liver fibrosis, and hepatocellular carcinoma. Immunization with one rVV strain (rVV-N25), which encoded nonstructural HCV proteins, suppressed serum inflammatory cytokine levels and alleviated the symptoms of pathological chronic hepatitis C within 7 days after injection. Furthermore, HCV protein levels in liver tissue also decreased in a CD4 and CD8 T-cell-dependent manner. Consistent with these results, we showed that rVV-N25 immunization induced a robust CD8 T-cell immune response that was specific to the HCV nonstructural protein 2. We also demonstrated that the onset of chronic hepatitis in CN2-29^(+/−)^/MxCre^(+/−)^ mice was mainly attributable to inflammatory cytokines, (tumor necrosis factor) TNF-α and (interleukin) IL-6. Thus, our generated mice model should be useful for further investigation of the immunological processes associated with persistent expression of HCV proteins because these mice had not developed immune tolerance to the HCV antigen. In addition, we propose that rVV-N25 could be developed as an effective therapeutic vaccine.

## Introduction

Hepatitis C virus (HCV) is a major public health problem; approximately 170 million people are infected with HCV worldwide [1]. HCV causes persistent infections that can lead to chronic liver diseases such as chronic hepatitis, liver cirrhosis, and hepatocellular carcinoma (HCC) [2]. Antiviral drugs are not highly effective in individuals with a chronic infection; furthermore, an effective vaccine against HVC has not been developed. A convenient animal model of HCV infection will greatly facilitate the development of an effective HCV vaccine.

Transgenic mice that express HCV proteins have been generated to study HCV expression [3], [4]; however, in each of these cases, the relevant transgenes is expressed during embryonic development; therefore, the transgenic mice become immunotolerant to the transgenic products, and consequently, the adult mice are not useful for investigations of the pathogenesis of chronic hepatitis C. To address this problem, we developed a system that can drive conditional expression of an HCV transgene; our system involves the Cre/*loxP* system and a recombinant adenovirus capable of expressing Cre recombinase [5], [6]. Concerns have been expressed that an adenovirus and transient expression of HCV proteins could induce immune responses [5] and, therefore, obscure any evidence of the effect of the host immune responses on chronic liver pathology. Therefore, here, we used a Cre/*loxP* switching system to generate an immunocompetent mouse model of HCV protein expression; with this system, we could study the host immune responses against HCV proteins.

Folgori et al. (2006) reported effective vaccination of chimpanzees with an adenoviral vector and plasmid DNA encoding the HCV nonstructural region. This technique protected the liver tissues from acute hepatitis, which results when whole animals are challenged with virus [7]. However, this vaccine has not yet been shown to be effective against chronic HCV infection.

Here, we aimed to address how HCV expression causes chronic liver diseases and to provide new options for HCV vaccine development. Using LC16m8, a highly attenuated strain of vaccinia virus (VV), we generated three recombinant vaccinia viruses (rVVs) that each encoded one of three different HCV proteins and found that one recombinant virus (rVV-N25), which encoded nonstructural HCV proteins, resolved pathological chronic hepatitis C symptoms in the liver. We also found that immunization with rVV-N25 suppressed HCV core protein levels in the livers of transgenic mice; moreover, this suppression was mediated by CD4 and CD8 T cells, as has been previously reported [8].

## Results

### Generation of a Model of Persistent HCV Protein Expression

**Figure 1:**
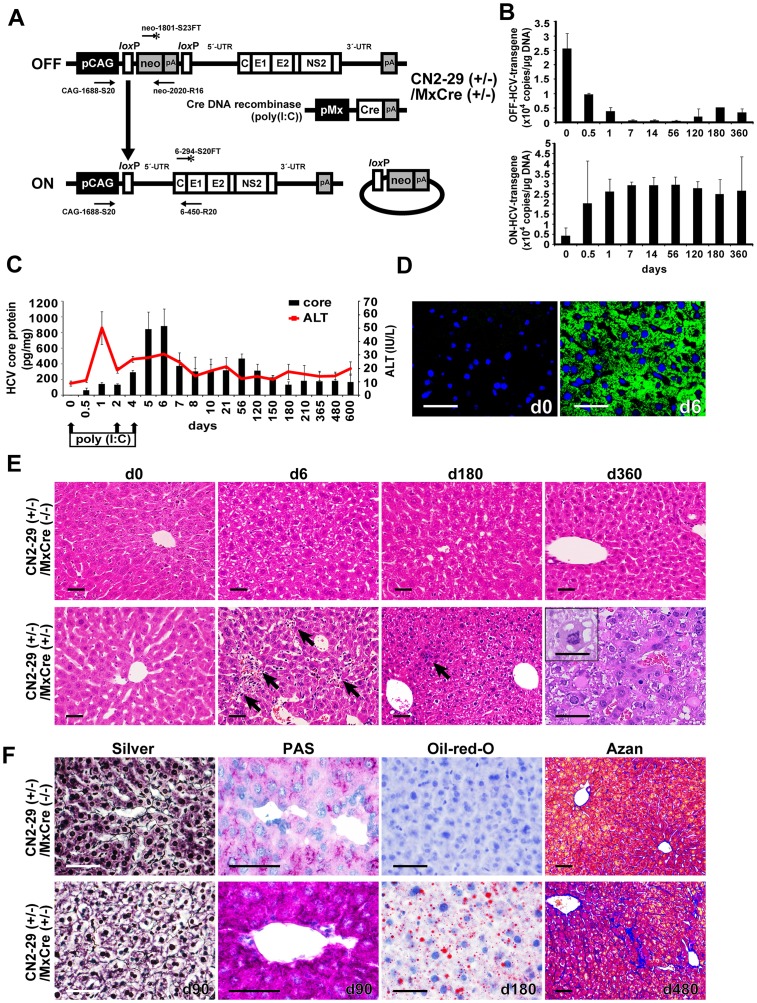
Pathogenesis in immunocompetent mice with persistent HCV expression. (**A**) Structure of CN2-29^(+/−)^/MxCre^(+/−)^ and the Cre-mediated activation of the transgene unit. R6CN2 HCV cDNA was cloned downstream of the CAG promoter, neomycin-resistant gene (*neo*), and poly A (pA) signal flanked by two *loxP* sequences. This cDNA contains the core, E1, E2, and NS2 regions. (**B**) Cre-mediated genomic DNA recombination. After poly(I:C) injection, genomic DNA was extracted from liver tissues and analyzed by quantitative RTD-PCR for Cre-mediated transgenic recombination. The transgene was almost fully recombined in transgenic mouse livers 7 days after the injection. In all cases, n = 3 mice per group. (**C**) HCV core protein expression was sustained for at least 600 days after poly(I:C) injection. (**D**) Immunohistochemical analysis revealed that most hepatocytes expressed the HCV core protein within 6 days after injection. (**E**) Liver sections from CN2-29^(+/−)^/MxCre^(+/−)^ mice after the poly(I:C) injection. Infiltrating lymphocytes (arrows) were observed on days 6 and 180; Hepatocellular carcinoma (HCC) was observed on day 360. In contrast, these pathological changes were not observed in CN2-29^(+/−)^/MxCre^(−/−)^ mice after the injection. The inset image shows abnormal mitosis in a tumor cell. (**F**) Hepatocyte swelling and abnormal architecture of liver-cell cords (silver staining), as well as abnormal glycogen accumulation (PAS staining) were observed on day 90 in CN2-29^(+/−)^/MxCre^(+/−)^ mice. We observed steatosis (oil-red-O staining) on day 180 and, subsequently, fibrosis (Azan staining) on day 480. The scale bars indicate 50 µm.

To produce adult mice that express an HCV transgene, we bred CN2-29 transgenic mice, which carry an HCV transgene, [5], [6], [9] with Mx1-Cre transgenic mice [10], which express Cre recombinase in response to interferon (IFN)-α or a chemical inducer of IFN-α, poly(I:C) (Figure 1A). Following poly(I:C) injection, the HCV transgene was rearranged, and HCV sequences were expressed in the livers of F1 progeny (CN2-29^(+/−)^/MxCre^(+/−)^ mice) within 7 days after poly(I:C) injection (Figure 1B).

To evaluate the characteristic features of these CN2-29^(+/−)^/MxCre^(+/−)^ mice, we analyzed serum alanine aminotransferase (ALT) and liver HCV core protein levels after poly(I:C) injection. As illustrated in Figure 1C, serum ALT levels increased and reached a peak at 24 h after the first poly(I:C) injection; this elevation appeared to be a direct result of the poly(I:C) treatment, which causes liver injury [11]. After this peak, serum ALT levels dropped continuously until day 4, and then ALT levels began to increase, as did HCV core protein levels. Thereafter, the HCV core protein was expressed consistently for at least 600 days.

Histological analysis showed HCV core protein expression in most hepatocytes of the transgenic mice; these mice showed evidence of lymphocytic infiltration that was caused by the HCV core proteins (Figure 1D and E). These observations, in addition to the modified histology activity index (HAI) scores, indicated that expression of HCV proteins caused chronic hepatitis in the CN2-29^(+/−)^/MxCre^(+/−)^ mice because a weak, though persistent, immune response followed an initial bout of acute hepatitis (Figure S1). Moreover, we observed a number of other pathological changes in these mice – including swelling of hepatocytes, abnormal architecture of liver-cell cords, abnormal accumulation of glycogen, steatosis, fibrosis, and HCC (Figures 1E and F, Table S1). Steatosis was mild in the younger mice (day 21) and became increasingly severe over time (days 120 and 180; Figure S2). Importantly, none of the pathological changes were observed in the CN2-29^(+/−)^/MxCre^(−/−)^ mice after poly(I:C) injection (Figure 1F).

### Recombinant Vaccinia Virus Immunization in HCV Transgenic Mice

**Figure 2:**
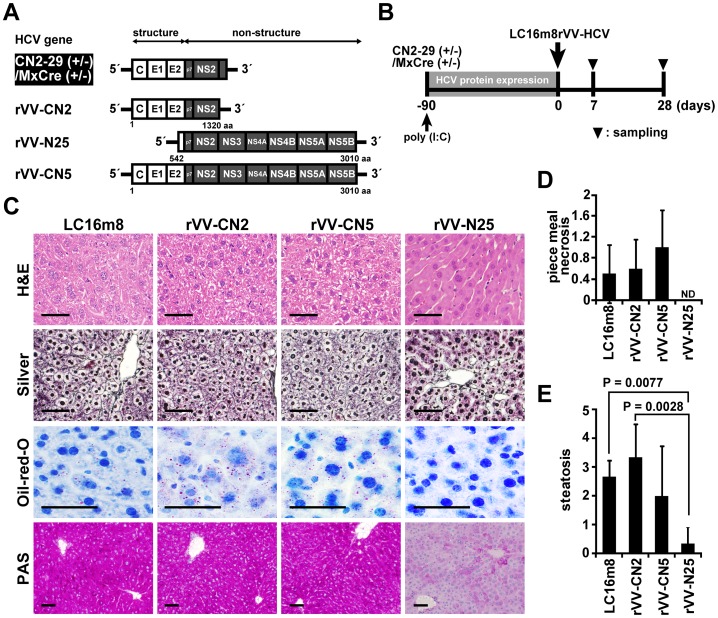
Effects of rVV-HCV treatment on the CN2-29^(+/−)^/MxCre^(+/−)^ mice. (**A)** HCV gene structure in the CN2-29^(+/−)^/MxCre^(+/−)^ mice and recombinant vaccinia viruses (rVV-HCV). MxCre/CN2-29 cDNA contains the core, E1, E2, and NS2 regions. The rVV-CN2 cDNA contains the core, E1, E2, and NS2 regions. The rVV-N25 cDNA contains the NS2, NS3, NS4A, NS4B, NS5A, and NS5B regions. The rVV-CN5 cDNA contains the entire HCV region. (**B**) Four groups of CN2-29^(+/−)^/MxCre^(+/−)^ mice were inoculated intradermally with rVV-CN2, rVV-N25, rVV-CN5, or LC16m8 90 days after the poly(I:C) injection. Blood, liver, and spleen tissue samples were collected 7 and 28 days after the inoculation. (**C**) Liver sections from the four groups of CN2-29^(+/−)^/MxCre^(+/−)^ mice 7 days after the inoculation. The sections were stained with H&E, silver, oil-red-O, or PAS. The scale bars indicate 50 µm. (**D**) Histological evaluation of piecemeal necrosis in the four groups of CN2-29^(+/−)^/MxCre^(+/−)^ mice 7 days after inoculation. (**E**) Histological evaluation of steatosis in the four groups of CN2-29^(+/−)^/MxCre^(+/−)^ mice 7 days after inoculation. Significant relationships are indicated by a P-value.

To determine whether activation of the host immune response caused the reduction with HCV protein levels in the livers of CN2-29^(+/−)^/MxCre^(+/−)^ mice, we used a highly attenuated VV strain, LC16m8, to generate three rVVs [12]. Each rVV encoded a different HCV protein; rVV-CN2 encoded mainly structural proteins, rVV-N25 encoded nonstructural proteins, and rVV-CN5 encoded the entire HCV protein region (Figure 2A). Because rVVs can express a variety of proteins and induce strong and long-term immunity, they have been evaluated as potential prophylactic vaccines [13].

We used western blots to confirm that each HCV protein was expressed in cell lines. Each of seven proteins – the core, E1, E2, NS3-4A, NS4B, NS5A, and NS5B – was recognized and labeled by a separate cognate antibody directed (Figure S3). To induce effective immune responses against HCV proteins in transgenic mice, we injected an rVV-HCV (rVV-CN2, rVV-CN5, or rVV-N25) or LC16m8 (as the control) intradermally into CN2-29^(+/−)^/MxCre^(+/−)^ mice 90 days after poly(I:C) injection (Figure 2B). Analysis of liver sections 7 days after immunization with rVV-N25 revealed dramatic improvement in a variety of pathological findings associated with chronic hepatitis – including piecemeal necrosis, hepatocyte swelling, abnormal architecture of liver-cell cords, abnormal accumulation of glycogen, and steatosis (Figures 2C–E). Collectively, these results demonstrated that only the rVV-N25 treatment resulted in histological changes indicative of improvement in the chronic hepatitis suffered by the transgenic mice.

To determine whether rVV-N25 treatment induced the same effect in other strains of HCV transgenic mice, we analyzed RzCN5-15^(+/−)^/MxCre^(+/−)^ mice, which express all HCV proteins; in these mice, chronic hepatitis was resolved within 28 days of immunization with rVV-N25. Taken together, these findings indicated that rVV-N25 had a dramatic therapeutic effect on both types of HCV transgenic mice (Figure S4).

### Treatment with rVV-N25 Reduced the HCV Core Protein Levels in the Livers

**Figure 3:**
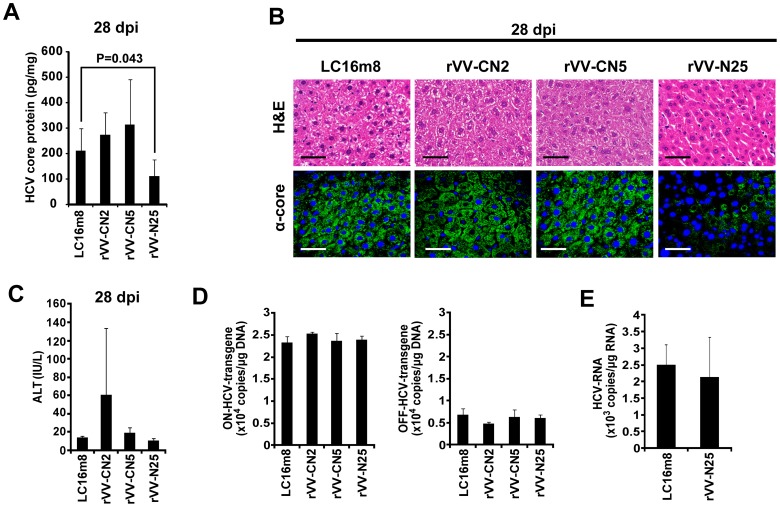
Effects of HCV core protein expression on the livers of CN2-29^(+/−)^/MxCre^(+/−)^ mice inoculated with rVV-HCV. (**A**) Expression of the HCV core protein in the four treatment groups of CN2-29^(+/−)^/MxCre^(+/−)^ mice 28 days after the inoculation. Significant relationships are indicated by a P-value. (**B**) H&E staining and immunohistochemical analysis for HCV core protein in the LC16m8-, rVV-CN2-, rVV-CN5-, or rVV-N25-treated CN2-29^(+/−)^/MxCre^(+/−)^ mice 28 days after the inoculation. Liver sections were stained with the anti-core monoclonal antibody. The scale bars indicate 50 µm. (**C**) Effects of HCV core protein expression on serum ALT levels in the four treatment groups of CN2-29^(+/−)^/MxCre^(+/−)^ mice 28 days after immunization. (**D**) Cre-mediated genomic DNA recombination in the four treatment groups 28 days after immunization. (**E**) Expression of HCV mRNA in the LC16m8- or rVV-N25-treated CN2-29^(+/−)^/MxCre^(+/−)^ mice 28 days after immunization. In all cases, n = 6 mice per group.

To assess in detail the effects of rVV-HCV immunization on HCV protein clearance from the livers of CN2-29^(+/−)^/MxCre^(+/−)^ mice, we monitored the levels of HCV core protein in liver samples via ELISA. We found that within 28 days after immunization the HCV core protein levels were significantly lower in livers of rVV-N25-treated mice than in those of control mice (Figure 3A). Immunohistochemical analysis indicated that, within 28 days after immunization, levels of HCV core protein were substantially lower in the livers of CN2-29^(+/−)^/MxCre^(+/−)^ mice than in those of control mice (Figure 3B). Importantly, neither resolution of chronic hepatitis nor reduction in the HCV protein levels was observed in the mice treated with LC16m8, rVV-CN2, or rVV-CN5. These results indicated that HCV non-structural proteins might be important for effects of therapeutic vaccines. In contrast, rVV-CN5 which encoded HCV structural and non-structural proteins did not show any significant effects. These results indicated that HCV structural proteins might have inhibited the therapeutic effects of the non-structural proteins. Therefore, it may be important to exclude the HCV structural proteins (aa 1–541) as antigenic proteins when developing therapeutic vaccines against chronic hepatitis C.

In addition, we measured serum ALT levels in CN2-29^(+/−)^/MxCre^(+/−)^ mice from all four treatment groups 28 days after rVV-HCV immunization. Serum ALT levels were not significantly different in the rVV-N25-treated mice and control mice (Figure 3C); this finding indicated that rVV-N25 treatment did not cause liver injury and that the antiviral effect was independent of hepatocyte destruction.

We hypothesized that the reduction in the levels of HCV core protein in rVV-HCV-treated mice was not caused by cytolytic elimination of hepatocytes that expressed HCV proteins. To investigate this hypothesis, we conducted an RTD-PCR analysis of genomic DNA from liver samples of CN2-29^(+/−)^/MxCre^(+/−)^ mice. The recombined transgene was similar in rVV-N25-treated and control mice 28 days after immunization (Figure 3D). We also measured the expression of HCV mRNA in LC16m8-treated CN2-29^(+/−)^/MxCre^(+/−)^ mice with that in rVV-N25-treated CN2-29^(+/−)^/MxCre^(+/−)^ mice 28 days after immunization; the HCV mRNA levels did not differ between rVV-N25-treated CN2-29^(+/−)^/MxCre^(+/−)^ and control mice (Figure 3E). These results indicated that rVV-N25-induced suppression of HCV core protein expression could be controlled at a posttranscriptional level.

### Role of CD4 and CD8 T cells in rVV-N25-treated Mice

**Figure 4:**
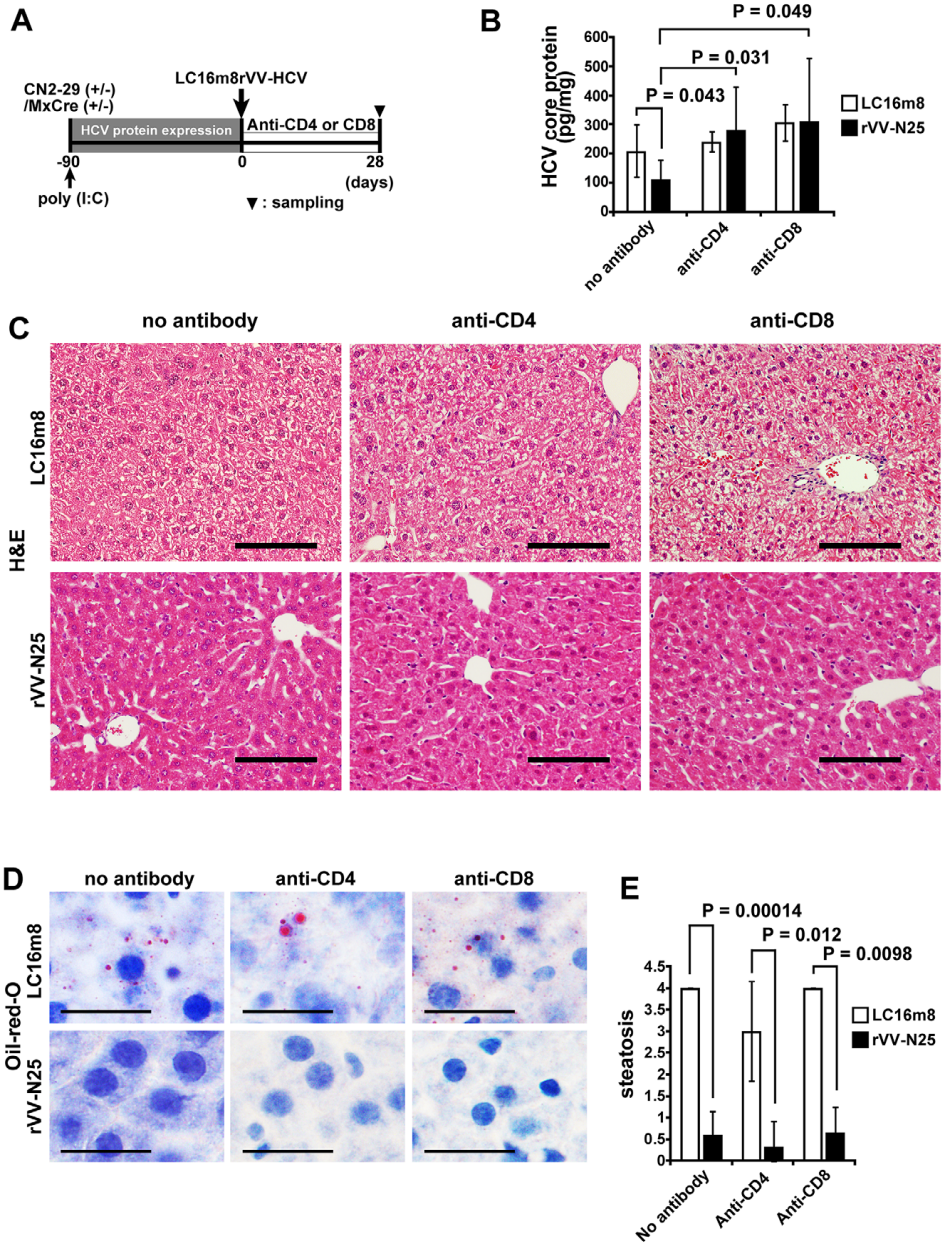
Role of CD4 and CD8 T cells in rVV-N25-treated mice. (**A**) Schematic diagram depicts depletion of CD4 and CD8 T cells via treatment with monoclonal antibodies. (**B**) Comparison of HCV core protein expression in control, CD4-depleted, and CD8-depleted mice 28 days after immunization with LC16m8 or rVV-N25. (**C, D**) Histological analysis of liver samples from CD4-depleted or CD8-depleted CN2-29^(+/−)^/MxCre^(+/−)^ mice 28 days after immunization with LC16m8 or rVV-N25. The scale bars indicate 100 µm (**C**) and 50 µm (**D**). (**E**) Histological evaluation of steatosis in liver samples from CD4-depleted or CD8-depleted CN2-29^(+/−)^/MxCre^(+/−)^ mice 28 days after immunization with LC16m8 or rVV-N25. Significant relationships are indicated by a P-value.

Viral clearance is usually associated with CD4 and CD8 T-cell activity that is regulated by cytolytic or noncytolytic antiviral mechanism [14]. To determine whether CD4 or CD8 T-cell activity was required for the reduction in HCV core protein levels in the livers of transgenic mice, we analyzed the core protein levels in CN2-29^(+/−)^/MxCre^(+/−)^ mice immunized with rVV-N25 in the absence of CD4 or CD8 T cells (Figure 4A). As expected, the mice lacking CD4 or CD8 T cells failed to show a reduction in HCV core protein levels (Figure 4B).

However, in mice lacking either CD4 or CD8 T-cells, the pathological changes associated with chronic hepatitis were resolved following rVV-N25 immunization, and the steatosis score of rVV-N25-treated mice was significantly lower than that of control mice (Figures 4C–E). These results indicated that CD4 and CD8 T cells were not responsible for the rVV-N25-induced amelioration of histological findings and that other inflammatory cell types may play an as-yet-unidentified role in the resolution of the pathological changes in these mice.

### rVV-N25 Immunization Induced an NS2-specific Activated CD8 T cells Response

Because we found that HCV protein reduction in the liver required CD8 T cells, we tested whether HCV-specific CD8 T cells were present in splenocytes 28 days after immunization. To determine the functional reactivity of HCV-specific CD8^+^ T cells, we performed a CD107a mobilization assay and intracellular IFN-γ staining. CN2-29 transgenic mice expressed the HCV structural protein and the NS2 region. However, rVV-N25 comprised only a HCV nonstructural protein. Thus, we focused on the role of the NS2 region as the target for CD8 T cells and generated EL-4 cell lines that expressed the NS2 antigen or the CN2 antigen.

Isolated splenocytes from immunized mice were co-cultured with EL-4CN2 or EL-4NS2 cell lines for 2 weeks and analyzed.

**Figure 5:**
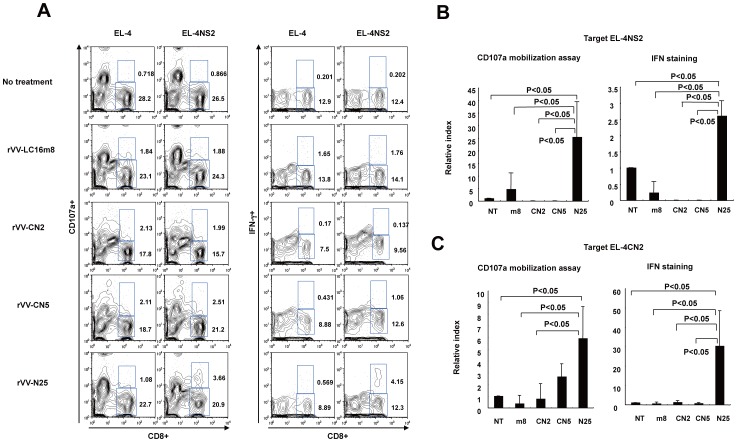
Immunization with rVV-N25 induced CD8 T-cell degranulation, a marker for cytotoxicity, and IFN-γ production. (**A**) The numbers represent the percentage of CD107a positive cells and negative cells (left two columns) and IFN-γ-positive cells and negative cells (right two columns). (**B, C**) The ratio of CD8^+^IFN-γ^+^ cells to all CD8 T cells for rVV-N25-treated mice was significantly higher than that for mice treated with any other rVV. Splenocytes (4 × 10^6^ per well) were cultured with EL-4CN2 or EL-4NS2 cell lines in RPMI 1640 complete medium including 3% T-STIM™ with ConA for 2 weeks. Harvested cells were incubated for 4 h with EL-4, EL-4CN2, or EL-4NS2 in combination with PE-labeled anti-CD107a mAb and monensin in RPMI 1640 complete medium with 50 IU/mL IL-2, according to the manufacturer's instruction. After incubation, cell suspensions were washed with PBS, and the cells were further stained with APC-labeled anti-IFN-γ mAb and Pacific blue-labeled anti-CD8 mAb. Harvested cells were stained with anti-CD107a-PE, anti-IFN-γ-APC, or anti-CD8-Pacific blue. Results that are representative of three independent experiments are shown. Significant relationships are indicated by P-value.

Cytolytic cell activation can be measured using CD107a, a marker of degranulation [15]. The ratio of CD8^+^CD107a^+^ cells to all CD8 T cells significantly increased in rVV-N25-treated splenocytes after co-culture with EL-4CN2 or EL-4NS2 (*P*<0.05), whereas splenocytes that had been treated with any other rVV were not detected (Figure 5A, B and C). These results indicated that rVV-N25 treatment increased the frequency of HCV NS2-specific activated CD8 T cells. Consistent with these results, the ratio of CD8^+^IFN-γ^+^ cells to all CD8 T cells for rVV-N25-treated mice was also significantly higher than that for mice treated with any other rVV (*P*<0.05). Taken together, these findings indicated that rVV-N25 induced an effective CD8 T-cell immune response and that NS2 is an important epitope for CD8 T cells.

### rVV-N25 Immunization Suppressed Inflammatory Cytokines Production

**Figure 6:**
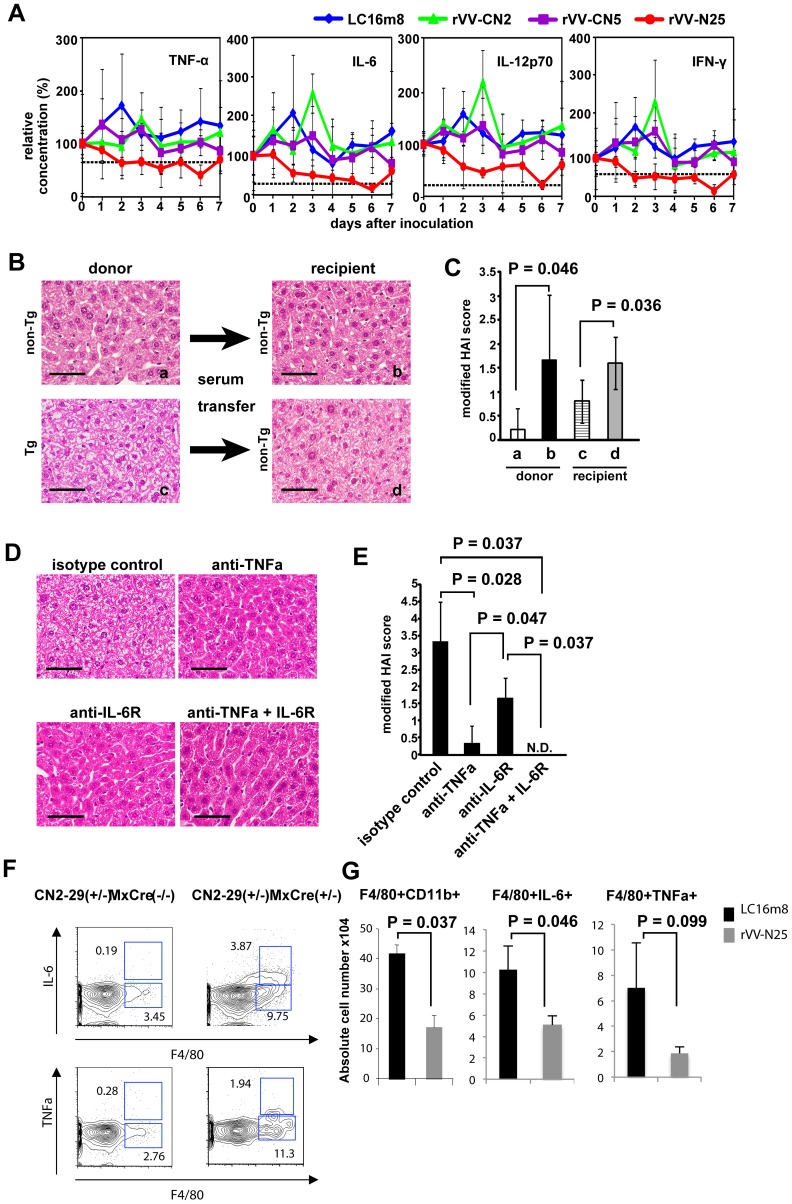
Immunization with rVV-N25 suppresses serum inflammatory cytokine levels. (**A**) Daily cytokine levels in the serum of CN2-29^(+/−)^/MxCre^(+/−)^ mice during the week following immunization with LC16m8, rVV-CN2, rVV-N25, or rVV-CN5. Values represent means ± SD (n  = 3) and reflect the concentrations relative to those measured on day 0. The broken lines indicate the baseline data from wild-type mice. In all cases, n  = 6 mice per group. (**B**) Liver sections from CN2-29^(+/−)^/MxCre^(+/−)^ and CN2-29^(+/−)^/MxCre^(−/−)^ mice. (**C**) Histology activity index (HAI) scores of liver samples taken from CN2-29^(+/−)^/MxCre^(+/−),^ or CN2-29^(+/−)^/MxCre^(−/−)^ mice. (**D**) Liver sections from CN2-29^(+/−)^/MxCre^(+/−)^ mice in which TNF-α was neutralized and the IL-6 receptor was blocked. The scale bars indicate 50 µm. (**E**) HAI scores of liver samples taken from CN2-29^(+/−)^/MxCre^(+/−)^ in which TNF-α was neutralized and the IL-6 receptor was blocked. Tg and non-Tg indicate CN2-29^(+/−)^/MxCre^(+/−)^ and CN2-29^(+/−)^/MxCre^(−/−)^, respectively. (F) Macrophages were the main producers of TNF-α and IL-6 in CN2-29^(+/−)^/MxCre^(+/−)^ mice following poly(I:C) injection. (G) Immunization with rVV-N25 reduced the number of macrophages in liver samples from CN2-29^(+/−)^/MxCre^(+/−)^ mice and suppressed TNF-α and IL-6 production from macrophages (Figure 6G). Significant relationships are indicated by a P-value.

To determine whether rVV-N25 treatment affected inflammatory cytokine production, we measured serum levels of inflammatory cytokines after rVV immunization. The serum levels of these inflammatory cytokines increased in the CN2-29^(+/−)^/MxCre^(+/−)^ mice (Figure 6A, Figure S5). Immunization with rVV-N25 affected serum levels of inflammatory cytokines in CN2-29^(+/−)^/MxCre^(+/−)^ mice and caused a return to the cytokine levels observed in wild-type untreated mice (Figure 6A). In wild-type mice, the cytokine levels remained unchanged after immunization (Figure 6A). These results indicated that inflammatory cytokines were responsible for liver pathogenesis in the transgenic mice.

To test the hypothesis that inflammatory cytokines were responsible for liver pathogenesis in CN2-29^(+/−)^/MxCre^(+/−)^ mice, we administered transgenic mouse serum intravenously into nontransgenic mice. We observed the development of chronic hepatitis in the nontransgenic mice within 7 days after the serum transfer (Figures 6B and C). This finding was consistent with the hypothesis that inflammatory mediators played a key role in inducing hepatitis. Furthermore, to investigate whether TNF-α and IL-6 played particularly critical roles in the pathogenesis of chronic hepatitis in the transgenic mice, we neutralized TNF-α and blocked the IL-6 receptor in the livers of these mice. As expected, chronic hepatitis did not develop in these mice. (Figure 6D and E).

Next, to determine which cell population(s) produced TNF-α, IL-6, or both during continuous HCV expression in CN2-29^(+/−)^/MxCre^(+/−)^ mice, we isolated intrahepatic lymphocytes (IHLs) and labeled the macrophages (the F4/80^+^ cells) with anti-TNF-α and anti-IL-6 antibodies using an intracellular cytokine detection method. Macrophages in CN2-29^(+/−)^/MxCre^(−/−)^ mice produced small amounts of TNF-α and IL-6, while those in CN2-29^(+/−)^/MxCre^(+/−)^ mice produced much larger amounts of these cytokines (Figure 6F).

Finally, we evaluated whether rVV-N25 treatment affected the number of macrophages, cytokine production by macrophages, or both; specifically, we isolated IHLs from CN2-29^(+/−)^/MxCre^(+/−)^ mice 7 days after immunization with rVV-N25 or with LC16m8. The percentage of macrophages (CD11b^+^F4/80^+^) among IHLs and IL-6 production from these macrophages were significantly lower in rVV-N25-treated mice than in control mice (Figure 6G). Though the percentage of TNF-α-producing macrophages was not significantly different in rVV-N25-treated and control mice (P = 0.099), rVV-N25 treatment appeared to suppress these macrophages. These results demonstrated that rVV-N25 had a suppressive effect on activated macrophages, and they indicated that this suppression ameliorated the histological indicators of chronic hepatitis.

## Discussion

Various HCV transgenic mouse models have been developed and used to examine immune response to HCV expression and the effects of pathogenic HCV protein on hepatocytes [4], [16], [17]. However, these transgenic mice develop tolerance to the HCV protein; therefore, examining immune response to HCV protein has been difficult.

To overcome the problem of immune tolerance in mouse models of HCV expression, we developed an HCV model in mice that relies on conditional expression of the core, E1, E2, and NS2 proteins and the Cre/loxP switching system [5], [6]; we showed that the injection of an Ad-Cre vector enhanced the frequency of HCV-specific activated CD8 T cells in the liver of these mice and caused liver injury. However, the Ad-Cre adenovirus vector alone causes acute hepatitis in wild-type mice. Nevertheless, the transgenic model was useful for evaluating interactions between the host immune system and viral protein (serum ALT level over 2,000 IU/L) [5]; HCV core protein levels were reduced and expression of this protein was transient (about 2 weeks). Therefore, this Ad-Cre-dependent model cannot be used to effectively investigate immune responses to chronic HCV hepatitis.

Here, we used poly (I:C)-induced expression of Cre recombinase to generate HCV transgenic mice in order to study the effect of HCV protein and confirmed that these mice developed chronic active hepatitis–including steatosis, lipid deposition, and hepatocellular carcinoma. These pathological findings in the transgenic mice were very similar to those in humans with chronic hepatitis C; therefore, this mouse model of HCV may be useful for analyzing the immune response to chronic hepatitis. However, experimental results obtained with this mouse model may not directly translate to clinical findings from patients with HCV infection because the expression of HCV proteins was not liver specific in these mice. Furthermore, poly(I:C) injection can activate innate immune responses and, consequently, might induce temporary liver injury [18]. Additionally, poly(I:C) injection has an adjuvant effect; specifically, it stimulates TLR3 signaling [19].

To evaluate whether poly(I:C) injection caused hepatitis in CN2-29^(+/−)^/MxCre^(−/−)^ mice, we examined serum ALT levels and liver histology following poly(I:C) injection. We found that, following poly(I:C) injection, serum ALT levels in CN2-29^(+/−)^/MxCre^(−/−)^ mice increased, reached a peak one day after injection, declined from day 1 to day 6, and were not elevated thereafter; this time-course indicated that poly(I:C) injection alone did not induced continuous liver injury (figure S6). Based on these findings, we believe that the effects of poly(I:C) injection in these mice did not confound our analysis of chronic hepatitis.

Immunization with rVV-N25 suppressed HCV protein levels in the liver, and this suppression was associated with ameliorated pathological chronic hepatitis findings (see Figure 3). Importantly, rVV-N25 treatment did not cause liver injury based on the serum ALT levels; therefore, this treatment was unlikely to have cytopathic effects on infected hepatocytes. These findings provided strong evidence that rVV-N25 treatment effectively halted the progression of chronic hepatitis. Immunization with plasmid DNA or with recombinant vaccinia virus can effectively induce cellular and humoral immune responses and exert a protective effect against challenge with HCV infection [20], [21]. However, findings from these previous studies revealed HCV immunization of both uninfected, naïve animals and immune-tolerant animals induced a HCV-specific immune response. In the model describe here; the animals were immune competent for HCV; therefore, our findings provided further important evidence that rVV-N25 was effective in the treatment of chronic hepatitis.

In addition, we demonstrated that rVV-N25 treatment in the absence of CD4 and CD8 T cells had no effect on HCV clearance. This important observation indicated that rVV-N25-induced HCV clearance was mediated by CD4 and CD8 T cells. Many studies have shown that spontaneous viral clearance during acute HCV infection is characterized by a vigorous, broadly reactive CD4 and CD8 T-cell response. [8], [22] HCV clearance and hepatocellular cytotoxicity are both mediated by CD8 antigen-specific (cytotoxic T lymphocyte) CTLs [23]. Consistent with these observations, rVV-N25 treatment effectively induced the accumulation of NS2-specific CD8 T cells, which express high levels of CD107a and IFN-γin the spleen. Notably, even with rVV-N25 immunization, the frequency of activated CD8 T cells was very low, and a minimum of 2-weeks incubation was required to distinguish the difference between rVV treatments. Even if a small population of specific CD8+ T cells played a relevant role in the reduction of core protein, it is difficult to assert that the only NS2-specific CD8+ T cells were important to this reduction. However, based on the results presented in Figure 4B, we are able to conclude that at least CD8+ and/or CD4+ T cells were important to the reduction in HCV core protein. Therefore, to elucidate the mechanism of HCV protein clearance, further investigation of not only the other T cell epitopes but also other immunocompetent cells is required.

Interestingly, rVV-N25 treatment–but not the rVV-CN2 or rVV-CN5 treatment–efficiently induced a HCV-specific activated CD8 T cells response; this difference in efficacy could have one or more possible causes. The HCV structural proteins (core, E1, and E2 proteins) in the rVV-CN construct may cause the difference; Saito et al. reported that injection with plasmid constructs encoding the core protein induced a specific CTL response in BALB/c mice [24]. Reportedly, CTL activity against core or envelope protein is completely absent from transgenic mice immunized with a plasmid encoding the HCV structural proteins, but core-specific CTL activity is present in transgenic mice that were immunized with a plasmid encoding the HCV core [21]. In contrast, when recombinant vaccinia virus expressing different regions of the HCV polyprotein were injected into BALB/c mice, only the HCV core protein markedly suppressed vaccinia-specific CTL responses [25]. Thus, the HCV core protein may have an immunomodulatory function [26]. Based on these reports and our results, we hypothesize that the causes underlying the effectiveness of rVV-N25 treatment were as follows: 1) this rVV construct included the core and envelope proteins and 2) the core protein had an immune-suppressive effect on CTL induction. Therefore, we suggest that exclusion of the core and envelope antigen as immunogen is one important factor in HCV vaccine design.

Interestingly, immunization with rVV-N25 rapidly suppressed the inflammatory response; however, immunization with either of the other rVVs did not (see Figure 6A). This result indicated that rVV-N25 may modulate inflammation via innate immunity, as well as via acquired immunity. Reportedly, Toll-like receptor (TLR)-dependent recognition pathways play a role in the recognition of poxviruses [27]. TLR2 and TLR9 have also been implicated in the recognition of the vaccinia virus [28], [29]. These findings indicate that TLR on dendritic cells may modulate the immunosuppressive effect of rVV-N25 in our model of HCV infection; however, further examination of this hypothesis is required. The finding that pathological symptoms in the HCV transgenic mice were completely blocked by intravenous injection of TNF-α and IL-6 neutralizing antibodies indicated that the progression of chronic hepatitis depended on inflammatory cytokines in serum, rather than the HCV protein levels in hepatocytes. Lymphocytes, macrophages, hepatocytes, and adipocytes each produce TNF-α and IL-6 [30], [31], and HCV-infected patients have elevated levels of TNF-α and IL-6 [32], [33]. Both cytokines also contribute to the maintenance of hepatosteatosis in mice fed a high-fat diet [34], and production of TNF-α and IL-6 is elevated in obese mice due to the low grade inflammatory response that is caused by lipid accumulation [35]. These findings indicate that both cytokines are responsible for HCV-triggered hepatosteatosis, and anti-cytokine neutralization is a potential treatment for chronic hepatitis if antiviral therapy is not successful.

The reduction of macrophages in number might be due to the induction of apoptosis by vaccinia virus *in vitro* infection as previous reported [36]. To understand the mechanisms responsible for the reduction of the number of macrophage, we performed another experiment to confirm whether the macrophages were infected with vaccinia virus inoculation. However, based on PCR analyses; vaccinia virus DNA was not present in liver tissue that contained macrophages (Figure S7). Furthermore, apoptosis of macrophages was not detected in liver samples (Data not shown). Based on these results, it is unlikely that the reduction in the number of macrophages was due to apoptosis induced by vaccinia virus infection. Although rVV-N25 reduced the number of macrophage, precise mechanism is still unknown. Further examination to elucidate the mechanism is required.

In conclusion, our findings demonstrated that rVV-N25 is a promising candidate for an HCV vaccine therapy. Additionally, the findings of this study indicate that rVV-N25 immunization can be used for prevention of HCV infection and as an antiviral therapy against ongoing HCV infection.

## Materials and Methods

### Ethics Statement

All animal care and experimental procedures were performed according to the guidelines established by the Tokyo Metropolitan Institute of Medical Science Subcommittee on Laboratory Animal Care; these guidelines conform to the Fundamental Guidelines for Proper Conduct of Animal Experiment and Related Activities in Academic Research Institutions under the jurisdiction of the Ministry of Education, Culture, Sports, Science and Technology, Japan, 2006. All protocols were approved by the Committee on the Ethics of Animal Experiments of the Tokyo Metropolitan Institute of Medical Science (Permit Number: 11–078). All efforts were made to minimize the suffering of the animals.

### Animals

R6CN2 HCV cDNA (nt 294–3435) [37] and full genomic HCV cDNA (nt 1–9611) [38], [39] were cloned from a blood sample taken from a patient (#R6) with chronic active hepatitis (Text S1). The infectious titer of this blood sample has been previously reported [40]. R6CN2HCV and R6CN5HCV transgenic mice were bred with Mx1-Cre transgenic mice (purchased from Jackson Laboratory) to produce R6CN2HCV-MxCre and R6CN5HCV-MxCre transgenic mice, which were designated CN2-29^(+/−)^/MxCre^(+/−)^ and RzCN5-15^(+/−)^/MxCre^(+/−)^ mice, respectively. Cre expression in the livers of these mice was induced by intraperitoneal injection of polyinosinic acid–polycytidylic acid [poly(I:C)] (GE Healthcare UK Ltd., Buckinghamshire, England); 300 µL of a poly(I:C) solution (1 mg/mL in phosphate-buffered saline [PBS]) was injected three times at 48-h intervals. All animal care and experimental procedures were performed according to the guidelines established by the Tokyo Metropolitan Institute of Medical Science Subcommittee on Laboratory Animal Care.

### Histology and Immunohistochemical Staining

Tissue samples were fixed in 4% paraformaldehyde in PBS, embedded in paraffin, sectioned (4-µm thickness), and stained with hematoxylin and eosin (H&E). Staining with periodic acid–Schiff stain, Azan stain, silver, or Oil-red-O was also performed to visualize glycogen degeneration, fibrillization, reticular fiber degeneration, or lipid degeneration, respectively.

For immunohistochemical staining, unfixed frozen liver sections were fixed in 4% paraformaldehyde for 10 min and then incubated with blocking buffer (1% bovine serum albumin in PBS) for 30 min at room temperature. Subsequently, the sections were incubated with biotinylated mouse anti-HCV core monoclonal antibody (5E3) for 2 h at room temperature. After being washed with PBS, the sections were incubated with streptavidin–Alexa Fluor 488 (Invitrogen). The nuclei were stained with 4',6-diamidino-2-phenylindole (DAPI). Fluorescence was observed using a confocal laser microscope (Laser scanning microscope 510, Carl Zeiss).

### Generation of rVVs

The pBR322-based plasmid vector pBMSF7C contained the ATI/p7.5 hybrid promoter within the hemagglutinin gene region of the vaccinia virus, which was reconstructed from the pSFJ1-10 plasmid and pBM vector [41], [42]. Separate full-length cDNAs encoding either the HCV structural protein, nonstructural protein, or all HCV proteins were cloned from HCV R6 strain (genotype 1b) RNA by RT-PCR. Each cDNA was inserted into a separate pBMSF7C vector downstream of the pBMSF7C ATI/p7.5 hybrid promoter; the final designation of each recombinant plasmid was pBMSF7C-CN2, pBMSF7C-N25, or pBMSF-CN5 (Figure 2). They were then transfected into primary rabbit kidney cells infected with LC16m8 (multiplicity of infection = 10). The virus–cell mixture was harvested 24 h after the initial transfection by scrapping; the mixture was then frozen at −80°C until use. The hemagglutinin-negative recombinant viruses were cloned as previously described [42] and named rVV-CN2, rVV-N25, or rVV-CN5. Insertion of the HCV protein genes into the LC16m8 genome was confirmed by direct PCR, and expression of each protein from the recombinant viruses was confirmed by western blot analysis. The titers of rVV-CN2, rVV-N25, and rVV-CN5 were determined using a standard plaque assay and RK13 cells.

### Statistical Analysis

Data are shown as mean ± SD. Data were analyzed using the nonparametric Mann–Whitney or Kruskal–Wallis tests or ANOVA as appropriate; GraphPad Prism 5 for Macintosh (GraphPad) was used for all analyses. *P* values *<*0.05 were considered statistically significant.

## Supporting Information

Figure S1 **HAI score of liver samples taken from CN2-29^(+/−)^/MxCre^(+/−)^ mice.**
(EPS)

Figure S2 **Lipid degeneration in samples of liver taken from CN2-29^(+/−)^/MxCre^(+/−)^ mice.**
(EPS)

Figure S3 **HCV protein expression after infection of LC16m8, rVV-CN2, rVV-N25, or rVV-CN5 into HepG2 cells.**
(EPS)

Figure S4 **Effects of treatment with rVV-N25 in RzCN5-15^(+/−)^/MxCre^(+/−)^ mice.**
(EPS)

Figure S5 **Daily cytokine profiles of the serum from CN2-29^(+/−)^/MxCre^(+/−)^ mice during the week following inoculation with LC16m8, rVV-CN2, rVV-N25, or rVV-CN5.**
(EPS)

Figure S6 **The immune response following poly(I:C) injection in the acute phase.**
(EPS)

Figure S7 **Detection of vaccinia virus DNA in the skin, liver, and spleen after inoculation with attenuated vaccinia virus (Lister strain) or highly attenuated vaccinia virus (LC16m8 strain).**
(EPS)

Table S1 **Incidence of hepatocellular carcinoma in male and female transgenic mice at 360, 480, and 600 days after poly(I:C) injection.**
(EPS)

Text S1 **Supporting information including material and methods, and references.**
(DOCX)
