# Supplementary material for: Immunization with a Recombinant Vaccinia Virus That Encodes Nonstructural Proteins of the Hepatitis C Virus Suppresses Viral Protein Levels in Mouse Liver
Source: PLoS One. 2012 Dec 17;7(12):e51656. doi: 10.1371/journal.pone.0051656 (PMC3524174; doi:10.1371/journal.pone.0051656)
Supplement: Text S1 — Supporting information including material and methods, and references. (DOCX) [file pone.0051656.s009.docx]

Material and methods

*Animals*

To create the HCV transgene, we used pCALNCN2/59-2, a plasmid that contains a expression-switching transgenic unit CALNCN2, which is similar in design to CALNLZ.[[1](#_ENREF_1)] CALNCN2 consists of the following components in the following order: a CAG promoter, a *loxP* sequence, the *neo* gene, the SV40 poly(A) signal, a second *loxP* sequence, the R6CN2 HCV cDNA, and the globin poly(A) signal. CALNCN2 was excised from pCALNCN2/59-2 by *Hin*dIII digestion and purified by gel electrophoresis and ultracentrifugation. Purified CALNCN2 was then microinjected into fertilized C57/BL6/cCrSlc mouse eggs (Japan SLC). Ear punch specimens were collected from potential founders for analysis of the presence of the transgene. Genomic DNA was isolated from each ear punch specimen and then used as a template for PCR amplification; HCV-specific primers (6-294-S20, 5′-TGATAGGGTGCTTGCGAGTG-3′ and 6-604-R18, 5′-TTGCCATAGAGGGGCCAA-3′) were also used.

*Assessment of Cre-mediated DNA recombination using liver samples from transgenic mice*

Liver samples from transgenic mice were incubated overnight at 37°C in lysis buffer (50 mM Tris-HCl [pH 8.0], 0.1 M NaCl, 20 mM EDTA, 1% SDS) that contained 1 mg/mL proteinase K. Total genomic DNA was then extracted from the tissue lysate using the phenol-chloroform extraction method. The copy number of HCV cDNA in each tissue sample was assessed by RTD-PCR[[2](#_ENREF_2)]; the probe was 6-294-S20FT (5′-[FAM]-TGATAGGGTGCTTGCGAGTG-[TAMRA]-3′), and the primer pair was CAG-1688-S20 (5′-GGTTGTTGTGCTGTCTCATC-3′) and 6-450-R20 (5′-ACAGGTAAACTCCACCAACG-3′). We generated a standard curve using pCALNCN2/59-2[[3](#_ENREF_3)] and performed RTD-PCR again with the probe, neo-1801-S23FT (5′-[FAM]-TCAAGAGACAGGATGAGGATCGT-[TAMRA]-3′), and primer pair, CAG-1688-S20 and neo-2020-R16 (5′-TGCCTCGTCCTGCAGT-3′). The *GAPDH* gene was used as an internal control for all samples. The analyses were conducted on an ABI PRISM 7700 system using the Universal PCR Master Mix (Applied Biosystems).

*Extraction of total RNA and quantitative RTD-PCR of HCV mRNA using liver samples from transgenic mice*

To measure the levels of HCV mRNA, we extracted total RNA samples from liver samples taken from transgenic mice; RNeasy mini kits were used for RNA extraction (Qiagen). The copy number of HCV mRNA in each liver sample was assessed by quantitative RTD-PCR using the 6-294-S20FT probe and the primer pair, CAG-1688-S20 and 6-450-R20. A standard curve was generated using pCALNCN2/59-2. Again, the *GAPDH* gene was used as an internal control for all the samples. The analyses were conducted on an ABI PRISM 7700 system using the Universal PCR Master Mix (Applied Biosystems).

*Biochemical analysis of mouse sera*

Sequential blood samples were obtained via orbital bleeding, and each serum sample was harvested by centrifugation of the respective blood at 10,000g for 3 min at 4°C. Serum ALT levels were measured using the Transaminase-CII Test A (Wako Pure Chemical Industries).

*Quantification of the HCV core protein in transgenic mouse livers*

Liver samples from transgenic mice were homogenized in 0.5 mL RIPA buffer and centrifuged at 10,000g for 10 min. Protein concentration in each supernatant was measured using the Bradford method (DC protein assay; Bio-Rad Laboratories). The concentration of the HCV core protein in liver samples was determined using the Ortho HCV Core Protein ELISA kit (Ortho-Chemical Diagnostics Japan).

*Histology*

Tissue samples were fixed in 4% paraformaldehyde in PBS, embedded in paraffin, sectioned (4-μm thickness), and stained with hematoxylin and eosin (H&E). Staining with periodic acid–Schiff stain, Azan stain, silver, or Oil-red-O was also performed to visualize glycogen degeneration, fibrillization, reticular fiber degeneration, and lipid degeneration, respectively.

For immunohistochemical staining, unfixed frozen liver sections were fixed in 4% paraformaldehyde for 10 min and then incubated with blocking buffer (1% bovine serum albumin in PBS) for 30 min at room temperature. Subsequently, the sections were incubated with biotinylated mouse anti-HCV core monoclonal antibody (5E3) for 2 h at room temperature. These sections were washed with PBS and then incubated with streptavidin–Alexa Fluor 488 (Invitrogen). The nuclei were stained with DAPI. Fluorescence was observed under a confocal laser microscope (Laser scanning microscope 510, Carl Zeiss).

*Histological evaluation*

The histological grade of hepatitis was determined using the HAI scoring system.[[4](#_ENREF_4)] The HAI score in each group was expressed as the average of the scores in five visual fields. The histological grade of steatosis was determined as follows: no visible fat, score 0; <5% of the liver infiltrated by fat, score 1; 5–25% fat, score 2; 25–50% fat, score 3; >50% fat, score 4.[[5-7](#_ENREF_5)].

*Cell isolation*

To isolate splenocytes, spleen samples were prepared by passing the tissues through a cell strainer (BD Bioscience). To isolate IHLs, single-cell suspensions were prepared from liver tissue perfused with PBS via the inferior vena cava. The perfused liver tissue samples were then digested in 10 ml RPMI 1640 (Nissui Pharmaceutical Co., Ltd.) containing 0.02% (w/v) collagenase IV (Invitrogen) and 0.002% (w/v) DNase I (Sigma-Aldrich) for 40 min at 37°C. The cell suspension was then layered over Lympholyte-M (Cedarlane) in PBS. After centrifugation for 20 min at 1000g rpm, IHLs were isolated at the interface.

***Adaptive transfer***

Transgenic mice were injected with poly(I:C); 90 days after these injections, we transferred 100 μL samples of serum from these transgenic mice into nontransgenic mice via the tail veins of the nontransgenic mice.

*Fluorescence-activated cell sorting (FACS) analysis*

To examine cytokine production, isolated splenocytes and IHLs were cultured *ex vivo* for 4 h in Brefeldin A (BD Bioscience). The cells were then stained with anti-CD3, anti-CD4, or anti-CD8 antibody (all from BD Bioscience). After fixation, the cells were incubated for 30 min in 25 μL PBS containing 0.5% saponin. Anti-mouse FITC-conjugated IFN-γ, granzyme B, or perforin (BD Bioscience) was added at a final dilution of 1:100, and the cells were incubated for 30 min at room temperature. The cells were washed and resuspended in 1 mL FACS buffer for analysis with the FACSCanto II system (BD Bioscience).

*Cells and viruses*

RK13 cells (ATCC: CCL-337) were cultured in MEM (Nissui Pharmaceutical Co., Ltd.) containing 5% fetal bovine serum (FBS). HepG2 cells (ATCC: HB-8065) were maintained in DMEM (Nissui Pharmaceutical) containing 10% FBS. To generate rVVs (strain LC16m8) that expressed HCV proteins, primary rabbit kidney cell cultures were prepared by overnight digestion of kidneys samples from 7-day-old inbred JW rabbits (Kitayama Labs). The kidney samples were digested with 100 PU/mL dispase (Sanko Junyaku Co., Ltd.), and the separated kidney cells were then grown in T175 flasks containing lactalbumin medium supplemented with Hank's salts (LH), 5% FCS, 100 U/mL penicillin, and 100 μg/mL streptomycin. When cell layers reached a confluence ~50%, the culture medium was replaced with lactalbumin medium containing Eagle's salts (LE), 5% FCS, 100 U/mL penicillin, and 100 μg/mL streptomycin. Both the rVV and LC16m8 strains were provided by the Chemo-Sero-Therapeutic Research Institute (Kumamoto, Japan).

*Western blotting for detection of HCV proteins*

HepG2 cells in collagen-coated, 6-well plates were infected with LC16m8, rVV-CN2, rVV-N25, or rVV-CN5 (MOI = 20). After 24 h, the cells were harvested, washed with PBS, and resolved in 100 μL RIPA buffer (10 mM Tris-HCl [pH 7.5], 0.15 M NaCl, 1% SDS, 0.5% Nonidet P-40, protease inhibitor cocktail [Complete; Roche Molecular Biochemicals]). The protein concentration in each cell lysate was measured using the Bradford method (DC protein assay; Bio-Rad Laboratories). The proteins in each cell lysate were resolved by SDS-polyacrylamide gel electrophoresis (SDS-PAGE) using Tris-glycine buffer, transferred to polyvinylidene difluoride (PVDF) membranes (GE Healthcare), activated with methanol, and blocked with 5% skim milk in PBS containing 0.1% Tween-20 (PBS-T). After washing with PBS-T, the protein-laden membranes were incubated overnight at 4°C in the presence of anti-core (mouse mAb; clone 31-2), anti-E1 (mouse mAb; clone 384), anti-E2 (mouse mAb; clone 544), anti-NS3-4A (mouse mAb; clone 10-1), anti-NS4B (mouse mAb; clone 52-1), anti-NS5A (rabbit pAb; provided by Dr. Matsuura, University of Osaka, Osaka, Japan), or anti-NS5B (mouse mAb; clone 14-5) antibodies with 5% skim milk in PBS-T. The membranes were then washed subsequently incubated with HRP-conjugated F(ab′)_2_ of anti-rabbit or anti-mouse IgG (GE Healthcare) for 1 h at room temperature. Protein expression levels were visualized using the ECL system (GE Healthcare) and a LAS3000 imager (Fujifilm).

*Immunization with rVV-HCV*

Following injection (90 days) with poly(I:C), transgenic mice were randomly placed into treatment groups and immunized intradermally with either LC16m8, m8rVV-CN2, m8rVV-N25, or m8rVV-CN5 at 1 × 10^8^ PFU/mouse.

*HCV-specific CTLs*

The spleens of rVV-immunized mice were harvested 28 days after immunization, and 4 × 10^6^ cells were co-cultured with EL-4 transformants (10^5^ cells) that expressed NS2. Co-culturing was performed in RPMI 1640 medium (Nissui Pharmaceutical Co., Ltd.) supplemented with streptomycin (100 μg/mL), penicillin (100 U/mL), HEPES (10 mM), sodium pyruvate (1 mM), 0.2% NaHCO_3_, glutamate (4 mM), 2-mercaptoethanol (5 μM), and 10% FBS in 24-well plates. The transformants were used as polyclonal effector cells after 5 days of *ex vivo* expansion. The percentage of NS2-specific positive cells [1] and the Relative index [2] were calculated using the following equations:

NS2-specific positive cells (%) = EL-4 NS2 (%) – EL-4 (%) [1]

Relative index = $\frac{NS2 specific positivie cells of Vaccinated group}{NS2 specific positive cells of No treatment group}$ $\frac{NS2 specific positivie cells of Vaccinated group}{NS2 specific positive cells of No treatment group}$ [2]

*Intracellular IFN-γ staining*

IHLs or splenocytes (5 × 10^5^) were cultured for 5 h in 96-well round-bottom plates containing 200 μL RPMI medium with either 5 × 10^5^ EL-4CN2 or E1N2 (E1, E2, and NS2) transformants or their respective parental cells. Human recombinant IL-2 (50 units) and 2 μL of BD GolgiPlug protein transport inhibitor (BD Bioscience) were added to each culture. For the macrophages experiments, LPS (Sigma) (0.5μg/mL) was added with BD GolgiPlug to the culture. After 4 h, the cells were harvested, washed in PBS containing 1% FCS, and incubated for 10 min on ice with unlabeled anti-mouse CD16/32 antibody (BD Bioscience) to block binding to Fc**γ**RII/III. The cells were surface-stained with Pacific Blue-conjugated monoclonal anti-mouse CD8 antibody (BD Bioscience), Pacific Blue-conjugated monoclonal anti-mouse CD11b antibody (BioLegend), and APCCy7-conjugated monoclonal anti-mouse F4/80 antibody (BioLegend) for 20 min on ice. The cells were washed to remove the unbound antibody, then we used a Cytofix/Cytoperm kit (BD Bioscience) to stain the cells with FITC-conjugated anti-mouse IFN-γ antibody, PE-conjugated anti-mouse TNF-α, and IL-6 (BD Bioscience). The cells were washed again and resuspended in 1 mL FACS buffer for analysis with the FACSCanto II system (BD Bioscience).

*CD107a mobilization assay*

To evaluate the cell-mediated cytotoxicity of HCV-specific CD8^+^ T cells, we performed a CD107a mobilization assay.[[8](#_ENREF_8)] This assay can be used for rapid assessment of cell-mediated cytotoxicity via sensitive detection of CD107a that becomes exposed on cell surfaced after antigen stimulation causes the secretion of the lytic granule contents such as perforin and granzymes. Splenocytes (4 × 10^6^ per well of a 24-well plate) were cultured with mitomycin C-treated EL-4CN2 or EL-4NS2 cell in RPMI 1640 complete medium that was supplemented with 3% T-STIM^TM^ without ConA (BD, NJ, USA) for 2 weeks. Harvested cells were incubated for 4 h with EL-4, EL-4CN2, or EL-4NS2 and PE-labeled anti-CD107a mAb (Biolegend) and the secretion inhibitor monensin in RPMI 1640 complete medium with 50 IU/mL IL-2, according to the manufacturer's instructions. After incubation, cell suspensions were washed with PBS, and the cells were further stained with APC-labeled anti-IFN-γ mAb and Pacific blue-labeled anti-CD8 mAb (Biolegend).

In vivo *depletion of CD4^+^ and CD8^+^ T cells*

CD4^+^ or CD8^+^ T-cell depletions were performed by inoculating mice intraperitoneally with 0.2 mL mouse ascites fluid containing by the GK1.5 (anti-CD4) or 53-6.72 (anti-CD8) monoclonal antibodies (both hybridomas were provided by Dr. S. Koyasu, Keio University, Tokyo, Japan). The monoclonal antibodies were first injected 6 days before the LC16m8 and rVV injections and then at 48-h intervals for 28 days.

*Neutralization of TNF-α and blockade of the IL-6 receptor*

TNF-α was neutralized by inoculating mice (via intraperitoneal injection) with 0.1 mg functional-grade rat anti-TNF-α antibody (clone MP6-XXT22; eBioscience). The monoclonal antibody or isotype control IgG was injected at 24-h intervals for 7 days. The IL-6 receptor was blocked by inoculating mice (via intraperitoneal injection) with 0.1 mg anti-IL-6 receptor antibody (Chugai Pharmaceutical Co., Ltd.). The monoclonal antibody or isotype control IgG was injected at 24-h intervals for 7 days.

*Cytokine and chemokine profiles*

We used Bio-Plex cytokine assay kits (Bio-Rad Laboratories) according to the manufacturer's instructions to measure the amounts of cytokines and chemokines in samples of mouse serum. Specifically, we used the Bio-Plex mouse cytokine 23-Plex Panel, which includes 23 cytokines (IL-1a, IL-1b, IL-2, IL-3, IL-4, IL-5, IL-6, IL-9, IL-10, IL-12 [P40], IL-12 [P70], IL-13, IL-17, Eotaxin, G-CSF, GM-CSF, IFN-γ, KC, MCP-1, MIP-1a, MIP-1b, RANTES, and TNF-α), and the Bio-Plex mouse cytokine 9-Plex Panel, which includes nine cytokines (IL-15, IL-18, FGF-basic, LIF, M-CSF, MIG, MIP-2, PDGF-β, and VEGF). The plates were then washed three times by vacuum filtration using 100 mL Bio-Plex wash buffer. Next, 25 μL of diluted detection antibody was added to each wells within plates, which was incubated with shaking for 30 min at room temperature. After three filter washes, 50 μL of streptavidin–phycoerythrin solution was added to each plate, which was then incubated with shaking for another 10 min at room temperature. Finally, they were washed by vacuum filtration three times, beads were suspended in Bio-Plex assay buffer, and samples were analyzed on a Bio-Rad 96-well plate reader using the Bio-Plex Suspension Array System and Bio-Plex Manager software (Bio-Rad Laboratories).

*Oligonucleotide primers for RTD-PCR of vaccinia virus genomic DNA*

RTD-PCR was performed using one of one set of PCR primers complementary to sequences located in the hemagglutinin (HA) gene of vaccinia virus genome region. PCR primers were universally conserved among four vaccinia virus strains (Lister, LC16m8, LC16mO, WR) known sequences. The primers located in the HA gene consisted of a forward primer pBSF110-350-R20: 5′-CTA GGA AAG ACA GCC ATA GC-3′ and a reverse primer HA-1-S: 5′- GGT CTT ATA TAC ACC GAGTAA GG-3′.

*Quantification of vaccinia virus genomic DNA by RTD-PCR*

C57/BL6/cCrSlc (Japan SLC) female mice (8 weeks old) were inoculated intradermally with either LC16m8, LC16m8rVV-N25, Lister strain at 1 x 10^8^ PFU/50 μl/mouse. 1, 3, 5, 7, 10, 14 days after the inoculation, skin, liver and spleen were cut into 3-5 mm^3^ pieces and immediately stored at -80°C until assay.

Vaccinia virus genomic DNA was extracted from liver tissue using proteinase K and sodium dodecyl sulfate (SDS), as follows. Liver tissue samples were lysed with 0.4-0.5 ml PK buffer (50 mM Tris-HCl pH 8.0, 20 mM EDTA, 0.1 M NaCl, 1% SDS, 1 mg/ml proteinase K) at 50°C for 12-14 hours and treated with 50 μg/ml RNase A at 37°C for 1 hour. Tissue chunks were spun out from the homogenized solution and 1 ml of the aqueous phase was extracted once with phenol, once with phenol-chloroform (1:1) and once with chloroform-isoamyl alcohol (24:1). The aqueous phase was precipitated with isopropanol and rinsed with 70% ethanol. Precipitated DNA was dissolved with TE (2 mM Tris-HCl pH8.0, 0.4 mM EDTA). Purified DNA solution was used for RTD-PCR, performed using a QuantiFast SYBR Green SYBR PCR kit with an CFX96 sequence detector system (Bio-Rad Laboratories).

Amplification reaction mixtures (25 μL) contained 100 ng/5 μL of DNA solution, 12.5 μL of 2 × QuantiFast SYBR Green SYBR PCR buffer, 1 μM of forward primer (pBSF110-350-R20) and 1 μM of reverse primer (HA-1-S). Thermal cycling conditions comprised: initial activation of the hot-start DNA polymerase at 95°C for 5 min. Subsequently, 50 cycles of amplification were performed at 95°C for 10 sec and 60°C for 30 sec. We subcloned a vaccinia virus hemagglutinin genome insert into pBMSF7c [[9](#_ENREF_9)]. Subcloned plasmid was purified and subsequently quantified by measuring optical density at 260 nm. The standard curve for this assay was calculated using a series of 10-fold dilutions of a vaccinia virus hemagglutinin genome subclned plasmid.

**References**

1. Kanegae Y, Lee G, Sato Y, Tanaka M, Nakai M, et al. (1995) Efficient gene activation in mammalian cells by using recombinant adenovirus expressing site-specific Cre recombinase. Nucleic Acids Res 23: 3816-3821.

2. Takeuchi T, Katsume A, Tanaka T, Abe A, Inoue K, et al. (1999) Real-time detection system for quantification of hepatitis C virus genome. Gastroenterology 116: 636-642.

3. Wakita T, Taya C, Katsume A, Kato J, Yonekawa H, et al. (1998) Efficient conditional transgene expression in hepatitis C virus cDNA transgenic mice mediated by the Cre/loxP system. J Biol Chem 273: 9001-9006.

4. Knodell RG, Ishak KG, Black WC, Chen TS, Craig R, et al. (1981) Formulation and application of a numerical scoring system for assessing histological activity in asymptomatic chronic active hepatitis. Hepatology 1: 431-435.

5. Baffy G, Zhang CY, Glickman JN, Lowell BB (2002) Obesity-related fatty liver is unchanged in mice deficient for mitochondrial uncoupling protein 2. Hepatology 35: 753-761.

6. Gilat T, Leikin-Frenkel A, Goldiner I, Juhel C, Lafont H, et al. (2003) Prevention of diet-induced fatty liver in experimental animals by the oral administration of a fatty acid bile acid conjugate (FABAC). Hepatology 38: 436-442.

7. Uesugi T, Froh M, Arteel GE, Bradford BU, Wheeler MD, et al. (2002) Role of lipopolysaccharide-binding protein in early alcohol-induced liver injury in mice. J Immunol 168: 2963-2969.

8. Burkett MW, Shafer-Weaver KA, Strobl S, Baseler M, Malyguine A (2005) A novel flow cytometric assay for evaluating cell-mediated cytotoxicity. J Immunother 28: 396-402.

9. Yasui F, Kai C, Kitabatake M, Inoue S, Yoneda M, et al. (2008) Prior immunization with severe acute respiratory syndrome (SARS)-associated coronavirus (SARS-CoV) nucleocapsid protein causes severe pneumonia in mice infected with SARS-CoV. J Immunol 181: 6337-6348.

**Supporting Information Legends**

**Figure S1.** HAI score of liver samples taken from CN2-29^(+/−)^/MxCre^(+/−)^ mice. In all cases, n = 3 mice per group.

**Figure S2.** Lipid degeneration in samples of liver taken from CN2-29^(+/−)^/MxCre^(+/−)^ mice. The Oil-red-O-positive lesions that appeared in the cytoplasm of hepatocytes after poly(I:C) injection indicated steatosis. The scale bar indicates 50 μm. In all cases, n = 3 mice per group.

**Figure S3.** HCV protein expression after infection of LC16m8, rVV-CN2, rVV-N25, or rVV-CN5 into HepG2 cells. LC16m8-infected HepG2 cells were used as a negative control. HCV structural proteins (core, E1, and E2) were detected in rVV-CN2-infected HepG2 cells. All HCV proteins were detected in rVV-CN5-infected HepG2 cells. HCV non-structural proteins (NS3-4A, NS4B, NS5A, and NS5B) were detected in rVV-N25-infected HepG2 cells.

**Figure S4.** Effects of treatment with rVV-N25 in RzCN5-15^(+/−)^/MxCre^(+/−)^ mice. **(A)** Structure of the RzCN5-15^(+/−)^/MxCre^(+/−)^ mouse transgene unit. **(B)** Liver sections of CN5-15^(+/−)^/MxCre^(+/−)^ mice after immunization. Infiltrating lymphocytes were observed in LC16m8-treated-RzCN5-15^(+/−)^/MxCre^(+/−)^ mice (indicated by arrows). The scale bar indicates 50 μm. **(C)** HAI score of liver samples taken from CN5-15^(+/−)^/MxCre^(+/−)^ mice after immunization. In all cases, n = 6 mice per group. Significant relationships are indicated by P-value.

**Figure S5.** Daily cytokine profiles of the serum from CN2-29^(+/−)^/MxCre^(+/−)^ mice during the week following inoculation with LC16m8, rVV-CN2, rVV-N25, or rVV-CN5. All values are relative to those measured at day 0. Broken lines indicate baseline values measured in wild-type mice. In all cases, n = 3 mice per group.

**Figure S6**. The immune response following poly(I:C) injection in the acute phase. (A) Liver sections from the CN2-29^(+/−)^/MxCre^(−/−)^ and CN2-29^(+/−)^/MxCre^(+/−)^ mice at 0, 1, 6, or 21 days after injection. Infiltrating lymphocytes were observed around portal tract (indicated by arrowheads). The scale bars indicate 100 µm. (B) Serum ALT levels following the poly(I:C) injection in CN2-29^(+/−)^/MxCre^(−/−)^ and CN2-29^(+/−)^/MxCre^(+/−)^ mice at 0, 1, 6, or 21 days after injection. Significant relationships (*P* < 0.05) are indicated by a P-value. In all cases, n = 3 mice per group.

**Figure S7**. Detection of vaccinia virus DNA in the skin, liver, and spleen after inoculation with attenuated vaccinia virus (Lister strain) or highly attenuated vaccinia virus (LC16m8 strain). Following rVV injection, vaccinia virus DNA was detected in the skin but not liver or spleen.

**Table S1.** Incidence of hepatocellular carcinoma in male and female transgenic mice at 360, 480, and 600 days after poly(I:C) injection.
